# The Importance of Cerebellar Connectivity on Simulated Brain Dynamics

**DOI:** 10.3389/fncel.2020.00240

**Published:** 2020-07-31

**Authors:** Fulvia Palesi, Roberta Maria Lorenzi, Claudia Casellato, Petra Ritter, Viktor Jirsa, Claudia A.M. Gandini Wheeler-Kingshott, Egidio D’Angelo

**Affiliations:** ^1^Department of Brain and Behavioral Sciences, University of Pavia, Pavia, Italy; ^2^Brain Connectivity Center, IRCCS Mondino Foundation, Pavia, Italy; ^3^Brain Simulation Section, Department of Neurology with Experimental Neurology, Charité – Universitätsmedizin Berlin and Berlin Institute of Health, Berlin, Germany; ^4^Bernstein Center for Computational Neuroscience, Berlin, Germany; ^5^Institut de Neurosciences des Systèmes – Inserm UMR1106, Aix-Marseille Université, Marseille, France; ^6^NMR Research Unit, Queen Square MS Centre, Department of Neuroinflammation, UCL Institute of Neurology, London, United Kingdom

**Keywords:** brain dynamics, The Virtual Brain, cerebro-cerebellar loop, multiscale approach, structural connectivity, functional connectivity

## Abstract

The brain shows a complex multiscale organization that prevents a direct understanding of how structure, function and dynamics are correlated. To date, advances in neural modeling offer a unique opportunity for simulating global brain dynamics by embedding empirical data on different scales in a mathematical framework. The Virtual Brain (TVB) is an advanced data-driven model allowing to simulate brain dynamics starting from individual subjects’ structural and functional connectivity obtained, for example, from magnetic resonance imaging (MRI). The use of TVB has been limited so far to cerebral connectivity but here, for the first time, we have introduced cerebellar nodes and interconnecting tracts to demonstrate the impact of cerebro-cerebellar loops on brain dynamics. Indeed, the matching between the empirical and simulated functional connectome was significantly improved when including the cerebro-cerebellar loops. This positive result should be considered as a first step, since issues remain open about the best strategy to reconstruct effective structural connectivity and the nature of the neural mass or mean-field models generating local activity in the nodes. For example, signal processing is known to differ remarkably between cortical and cerebellar microcircuits. Tackling these challenges is expected to further improve the predictive power of functional brain activity simulations, using TVB or other similar tools, in explaining not just global brain dynamics but also the role of cerebellum in determining brain states in physiological conditions and in the numerous pathologies affecting the cerebro-cerebellar loops.

## Introduction

The brain is made of several interconnected networks that differently contribute to generate its global activity. To improve the understanding of mechanisms that subtend physiological and pathological dynamics, these networks need to be investigated at different organization scales. Thus, a multiscale approach that combines results from microscopic, mesoscopic and macroscopic experiments could help facing the challenge. The relationship between brain structure, function and dynamics can be investigated using appropriate experimental and modeling approaches. The microscale concerns local neuronal microcircuits (e.g., a cerebral cortical microcolumn or a cerebellar cortical microzone), the mesoscale is a collection of local microcircuits possibly of different nature (e.g. a cortical area connected to the corresponding thalamic nucleus or a cerebellar microcomplex including multiple microzones and the connected deep cerebellar nuclei neurons), while the macroscale refers to large-scale circuits (e.g., the cerebral cortical and subcortical circuits forming cerebro-cerebellar loops). To date, microscale and mesoscale data have been made available for the rodent brain and are being used to implement detailed computational models and simulate the underlying physiological processes and computational rules (Markram et al., 2015; D’Angelo et al., 2016; Casali et al., 2019). Non-invasive functional macroscale data, in humans *in vivo*, can be acquired using electroencephalography (EEG) or magnetoencephalography (MEG), but magnetic resonance imaging (MRI) is the most widely used techniques for its great versatility.

Magnetic resonance imaging can provide several parameters informing about both structural and functional features of the brain. On the one hand, microstructural information can be inferred from diffusion-weighted (DW) MRI, which can be exploited for tractography, providing the only way of reconstructing *in vivo* axonal tracts (∼2 mm resolution) connecting distant brain regions. On the other hand, functional MRI (fMRI), by means of the blood oxygenation level dependent (BOLD) signal changes, reveals brain activity at very-low frequency (delta-band or below) providing an indirect measurement of the ensemble activity ongoing at cellular level. It should be noted that functional brain activity can be recorded with higher temporal resolution and better neuronal correlations using EEG or MEG, but these techniques are essentially used to reveal cerebral cortical signals. The rich information provided by MRI can also be exploited to calculate the brain connectome, i.e., a matrix of functional or structural connectivity between pairs of regions of gray matter. Once again, these connectomes have mostly been created for the cerebrum without including the cerebellum (Uddin et al., 2019; Kaestner et al., 2020).

The cerebellum is well known for its fundamental role in sensorimotor control, planning, and learning. Interestingly, evidence is growing on its role also in cognitive and emotional functions (D’Angelo, 2019). Given these functional implications and its extended connectivity with the cerebral cortex, it is probable that the cerebellum takes part in global brain dynamics. This aspect, however, remains largely unexplored. Recently, using advanced tractography, it has been possible to reconstruct the tracts wiring the cerebral cortex and the cerebellum demonstrating the existence of cerebro-cerebellar loops involving the associative areas (Palesi et al., 2015, 2017b). Moreover, resting-state fMRI (rs-fMRI) has demonstrated that the cerebellum is entrained into large-scale coherent oscillations together with cerebral cortical regions to form several resting-state networks, including the default-mode network, salience network and attention network in addition to the sensorimotor network (Buckner, 2013; Castellazzi et al., 2018). Finally, task-dependent fMRI has shown cerebellar activation, together with several cerebral cortical areas, during the execution of motor and cognitive tasks (Casiraghi et al., 2019). These data add to pioneering MEG recordings showing that the cerebellum is entrained into low-frequency oscillations during motor planning and execution (Gross et al., 2001). It is therefore imperative to consider the cerebellum in current and future studies combining whole-brain functional and structural connectivity with models to simulate brain dynamics.

One of such comprehensive frameworks making use of large scale data is The Virtual Brain (TVB) (Sanz Leon et al., 2013), which has recently been developed to simulate whole-brain dynamics in response to the challenge of bridging the gap between microscale, mesoscale and macroscale data. To do so, TVB comprises several tools that combine MRI brain data with mathematical data-driven models emulating cellular microcircuits behavior. Different brain regions are remapped onto nodes and wired through a subject-specific structural connectome (SC) obtained from DW MRI (Schirner et al., 2015; Proix et al., 2016), while brain dynamics are simulated using mathematical models [i.e., mean fields or neural masses (Pinotsis et al., 2014)] that can reproduce excitatory and inhibitory processes within a node. The resulting simulated neural activity can be converted into a functional connectome (FC) that can then be compared with the empirical functional data to assess the predictive power of the model. A few studies have demonstrated the potential of TVB to investigate physiological brain states not only in healthy subjects (Schirner et al., 2018) but also in neurological diseases. For example, in tumors and epilepsy TVB has been used to predict the surgical outcome, while in Alzheimer’s disease has been used to predict neurodegenerative progression (Aerts et al., 2018, 2020; Zimmermann et al., 2018; An et al., 2019).

Although the investigation of brain dynamics using combined experimental and modeling approaches is in rapid expansion, there are still major aspects worth being considered. Primary to our view is that only cerebral nodes and their connections are currently considered, while the cerebellum is overlooked. The second issue concerns the inaccuracy of SC, which includes false positive connections (Daducci et al., 2015; Jeurissen et al., 2019) and may propagate over simulated brain dynamics. Last, but quite relevant, TVB assumes that the neural mass and mean field models are the same for all nodes, although some initial works using different modalities have already addressed the need for heterogenous parameterization (Stefanovski et al., 2019) and for dedicated models for deep gray matter (GM) structures (van Wijk et al., 2018; Friston et al., 2019; Palesi et al., in press).

The cerebellum is tightly interconnected with the cerebral cortex and engages complex local circuits containing more than 50% of all brain neurons, suggesting the importance to assess its impact on whole-brain dynamics. Here, we wired cerebellar to cerebral nodes using TVB. Then, the cerebellar contribution was assessed in networks of different complexity to provide an initial evaluation of the impact of SC accuracy on simulated whole-brain dynamics. Moreover, we compared the contribution of cerebellar nodes either within the whole-brain network or within specific subnetworks. Ultimately, the pipeline presented here might be applied within TVB or adapted to other modeling frameworks, like Dynamic Causal Modeling (DCM) (Friston et al., 2003, 2019) that has been already used to address the contribution of cerebro-cerebellar loops in social mentalizing (Van Overwalle et al., 2019), to investigate the numerous neurological disorders affecting the cerebro-cerebellar loops, from cerebellar ataxia to autism, neurodegenerative disease, and multiple sclerosis (Jeong et al., 2012; Castellazzi et al., 2014; Palesi et al., 2018; Parmar et al., 2018; Farinelli et al., 2020). This modeling approach will prove especially useful to investigate brain rewiring and compensatory plasticity, which strongly depend on the cerebellum.

## Materials and Methods

**FIGURE 1:**
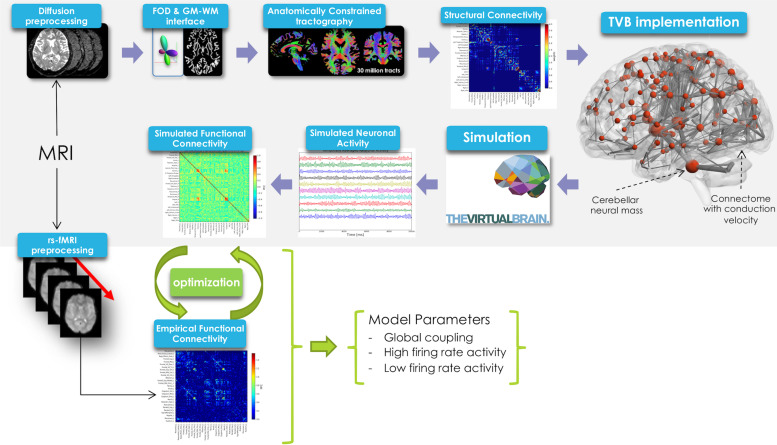
Schematic representation of the experimental and modeling workflow. From top left, clockwise: diffusion preprocessing, fiber orientation distribution calculation, whole-brain tractography, creation of structural connectome and its integration in TVB, spatio-temporal neural mass activity simulation, simulated functional connectivity matrix definition. Simulated FC was optimized using a model inversion approach with the empirical FC as target.

In this work TVB was used to simulate brain dynamics based on a Human Connectome Project (HCP) MRI dataset including DW and rs-fMRI data. The methodological workflow is shown in Figure 1.

### MRI Dataset

Minimally pre-processed MRI data were downloaded from ConnectomeDB^1^ (Van Essen et al., 2013). The dataset included high-quality DW data (1.25 mm isotropic resolution, *b* = 1000, 2000, 3000 s/mm^2^, 90 isotropically distributed directions/*b*-value and 18 b0 images), high-quality rs-fMRI data (2 mm isotropic resolution, TR/TE = 720/33.1 ms, 1200 volumes) and 3DT1-weighted images (0.7 mm isotropic resolution resampled at the same resolution of DW data) of 10 healthy subjects [3 males/7 females; 22–35 years (30.6 ± 4.1 years)] acquired using a customized Siemens 3T Connectome Skyra scanner with a 32-channel receive head coil.

### Definition of *ad hoc* Brain Atlas

An *ad hoc* atlas comprising 126 regions was created in MNI152 space. A total of 93 cerebral parcellations were defined combining (1) cortical regions from the Automated Anatomical Labeling (AAL) template (Tzourio-Mazoyer et al., 2002), (2) deep GM structures identified with FIRST (FMRIB Software Library, FSL^2^), while 33 cerebellar parcellations corresponded to those identified by the SUIT (A spatially unbiased atlas template of the cerebellum and brainstem) template (Diedrichsen et al., 2009). The atlas was transformed to subject-space (DW atlas) by inverting the calculated non-affine registration from DW to MNI152 space.

### Definition of Structural Connectivity and Lengths Matrices

3DT1-weighted images were segmented (FSL) in white matter (WM), GM, subcortical GM (Patenaude et al., 2011), and cerebrospinal fluid (CSF). From DW data, fiber orientation distributions were calculated separately for each tissue with the multi-shell multi-tissue constrained spherical deconvolution algorithm (Jeurissen et al., 2014) in MRtrix3^3^. Whole-brain Anatomically Constrained Tractography (Smith et al., 2012) was performed with 30 million streamlines and using probabilistic streamline tractography (iFOD2) (Tournier et al., 2010). To correct for spurious ipsilateral cerebro-cerebellar tracts, contralateral efferent and afferent cerebellar connections were selected from whole-brain tractograms as described in a previous work (Palesi et al., 2017a).

**FIGURE 2:**
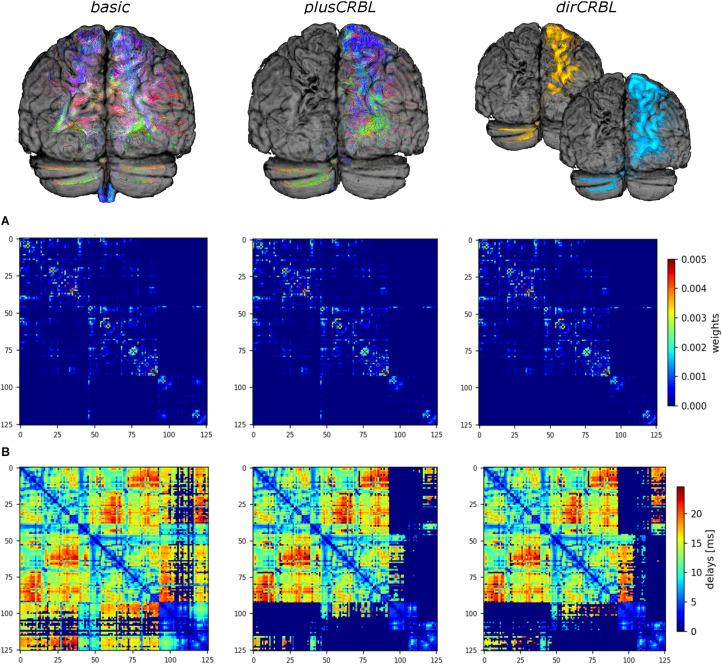
Structural connectome (SC) and time delay matrices. The top row shows the cerebro-cerebellar connectivity considered in each SC construct. For clarity only tracts connected to the left hemisphere of the cerebellum are shown. From left to right: *basic* (all tracts, including the spurious ones), *plusCRBL* (contralateral tracts only), *dirCRBL* (directional contralateral tracts). In *basic* and *plusCRBL* cerebellar connections are color coded with standard tractography rules (i.e., red for streamlines following left-to-right direction, green for anterior-posterior, and blue for superior-inferior), while, in the *dirCRBL*, yellow refers to efferent cerebellar connections and light blue refers to afferent ones to highlight anatomically inferred directionality. The middle row **(A)** shows the structural connectivity matrices, while the bottom row **(B)** shows the time delay matrices. All matrices refer to an SC construct in a randomly chosen subject.

In order to assess the impact of cerebellar connectivity and SC accuracy on simulated brain dynamics, three different subject-specific SC matrices (“SC constructs”) were generated. Whole-brain tractography and DW atlas were combined to extract streamlines (edges) between brain regions (nodes) and considering: (1) all cerebro-cerebellar tracts, as extracted from the whole-brain tractogram (*basic*); (2) contralateral cerebro-cerebellar tracts only, obtained by manually selecting the contralateral connections between cerebellum and cerebrum (*plusCRBL*); (3) directional contralateral cerebro-cerebellar tracts, obtained by extracting streamlines with the correct assumptions on the directional connectivity of cerebellar efferent streamlines, via the superior cerebellar peduncle, and cerebellar afferent streamlines, through the middle cerebellar peduncle (*dirCRBL*). The weight of each edge was normalized with respect to the total number of streamlines belonging to each network (*basic*, *plusCRBL*, *dirCRBL*). A corresponding matrix with edges weighted by the mean path length of each connection was also calculated. An example of these matrices is shown in Figure 2.

### Definition of Empirical FC Matrix

Resting-state fMRI data were preprocessed, realigned to the MNI152 template and noise components were removed (FIX, FSL) (Griffanti et al., 2014). Further steps were performed with CONN^4^ : 3DT1-weighted images were aligned to rs-fMRI data and segmented, then BOLD signal of each voxel was cleaned for the effect of any possible confound, including motion, physiological and other noise sources (i.e., BOLD signal from the WM and CSF) by applying a linear regression, a linear detrending and a band-pass filter with a window of (0.008–0.09 Hz).

Connectivity measures were calculated by extracting the average time-course per brain region (node) and correlating time-courses between pair of nodes. The resulting Fisher z-transformed coefficients defined the FC matrix, which was thresholded at 0.1206 to obtain the final empirical FC (empFC) matrix including cerebellar regions.

### Brain Dynamics Simulation With TVB

The Virtual Brain modeling consists of a series of steps: (1) definition of the long-range macroscopic SC; (2) selection of the mathematical model and parameters describing local (i.e., intra-nodal) GM functional dynamics; (3) simulation of the BOLD signal per node to generate the simulated FC matrix (simFC); (4) iterative tuning of model parameters to achieve the best matching between the simFC and empFC matrix; (5) simulation of brain dynamics with the optimal model parameters (for more details, see Ritter et al., 2013; Sanz-Leon et al., 2015). Here, these steps were repeated for each one of the 3 SC constructs.

Long-range SC was defined by the subject-specific SC matrix calculated from DW MRI. A reduced Wong-Wang model (Wong and Wang, 2006) was chosen to generate the neural activity S_*i*_ per node *i*:

$$\frac{\text{d}\,S_{i}}{\text{d}\,t}=\frac{-S_{i}}{\tau_{s}}+\gamma \,\left(1-S_{i}\right)\,H\,\left(x_{i}\right)+\sigma \,\eta_{i}\,\left(t\right)$$

$$H\,\left(x_{i}\right)=\frac{a\,x_{i}-b}{1-\text{exp}\,\left(-d\,\left(a\,x_{i}-b\right)\right)}$$

$$x_{i}=w\,J_{N}\,S_{i}+J_{N}\,G_{\text{coupl}}\,\sum_{j}C_{i\,j}\,S_{j}+I_{0}$$

where *H*(*x*_*i*_) is the transfer function that converts the input synaptic activity *x*_*i*_ into an output population firing rate, *C*_*ij*_ are the edges of SC matrix (between nodes *i* and *j*) reweighted by the global coupling parameter *G*_*coupl*_, η*_*i*_* (*t*) is a Gaussian white noise with amplitude σ (the other parameters are defined in Table 1). Conduction velocity (i.e., the speed of signal transfer along axonal tracts) was set to 10 m/s on the bases of physiological evidence (Purves et al., 2001), and time delays per edge were calculated on the basis of the tract length matrix (Figure 2). A model inversion approach was used to iteratively tune *G*_*coupl*_ across the whole-brain network in order to find the optimal *G*_*coupl*_ value that maximizes the matching of simFC with the empFC of all nodes. The physiological consistency of this procedure was evaluated calculating high and low firing rates (*H*_*high*_ and *H*_*low*_) at the optimal *G*_*coupl*_ value. Using these optimal parameters, subject-specific brain dynamics were simulated for a period of 6 min and a specific time-series per node and simFC were provided (Figure 1).

**TABLE 1:** Default reduced Wong-Wang model parameters.

Model parameter	Value	Unit	Description
a	270	nC^–1^	Sigmoid function parameters
b	108	Hz	
d	0.154	s	
γ	0.000641	–	Kinetic parameter
σ	0.001	nA	Amplitude of the Gaussian white noise η_*i*_(t)
w	1	–	Local excitatory recurrence
τ	100	ms	Kinetic parameter (NMDA decay time constant)
J_*N*_	0.2609	nA	Synaptic coupling
I_0_	0.3	nA	Effective external input

*Parameters of the reduced Wong-Wang model used in all simulations.*

### Integration of Cerebro-Cerebellar Connectivity in TVB

**FIGURE 3:**
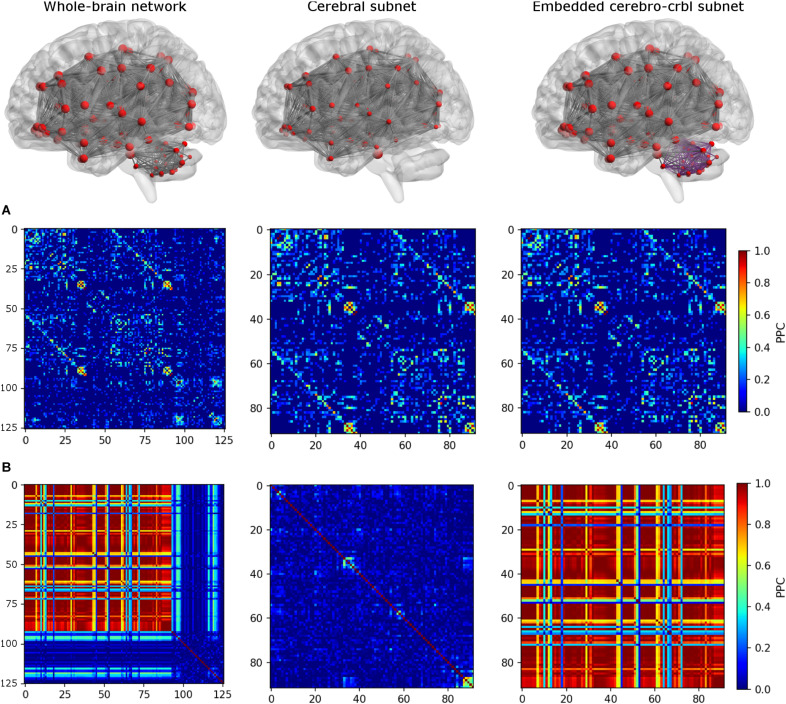
Functional connectivity (FC) matrices for each network. The top row shows the connections of each network. From left to right: *whole-brain network, cerebral subnetwork, embedded cerebro-cerebellar subnetwork*. Nodes are represented in red, while edges connecting nodes for which simulated FC (simFC) was calculated are in gray. In the *embedded cerebro-cerebellar subnetwork*, cerebellar connections are represented in purple. The middle row **(A)** shows the empirical FC, while the bottom row **(B)** shows simFC matrices for each network. All matrices are taken from a randomly chosen subject. For simplicity, only the structural connectome (SC) construct that considers appropriate cerebro-cerebellar connections (i.e., the case that we named *plusCRBL*) is shown, since the trend is the same in all SC constructs.

To investigate the impact of cerebellum on brain dynamics, three different networks were considered (see an example in Figure 3):

1. *Whole-brain network*: TVB simulation was run considering the whole-brain SC, and the simFC was derived for all brain nodes.
2. *Cerebral subnetwork*: TVB simulation was run considering only the cerebral SC, and the simFC was derived only for the cerebral nodes.
3. *Embedded cerebro-cerebellar subnetwork*: the simFC was evaluated only for the cerebral nodes as the discrepancy between simFC of (1) and (2).

For each of these three networks, *similarity* was evaluated by calculating the mean Pearson correlation coefficient (PCC) between empFC and simFC matrices. Furthermore, to assess the impact of SC *accuracy*, which is mainly affected by false positives, the three simFC described above and related *similarity* were calculated for each of the three SC constructs (*basic*, *plusCBL*, and *dirCBL*).

### Statistics

Statistical tests were performed using SPSS software version 21 (IBM, Armonk, New York, United States). All data were normally distributed (Shapiro–Wilk test), thus a general linear model with repeated measures was performed twice to assess all significant differences.

Firstly, the effect of the cerebellum on brain dynamics was assessed by comparing individual mean PCC between the three networks within each SC construct.

Secondly, the effect of SC accuracy was assessed by comparing between different SC constructs: (i) optimal *G*_*coupl*_, and *H*_*low*_ and *H*_*high*_ parameters; (ii) individual mean PCC for each network.

## Results

Results were obtained following the workflow shown in Figure 1. SC and time delays matrices are shown in Figure 2. SimFC and empFC are compared in Figure 3 for the three networks, i.e., *whole-brain network, cerebral subnetwork, embedded cerebro-cerebellar subnetwork*.

### Parameter Estimation

**FIGURE 4:**
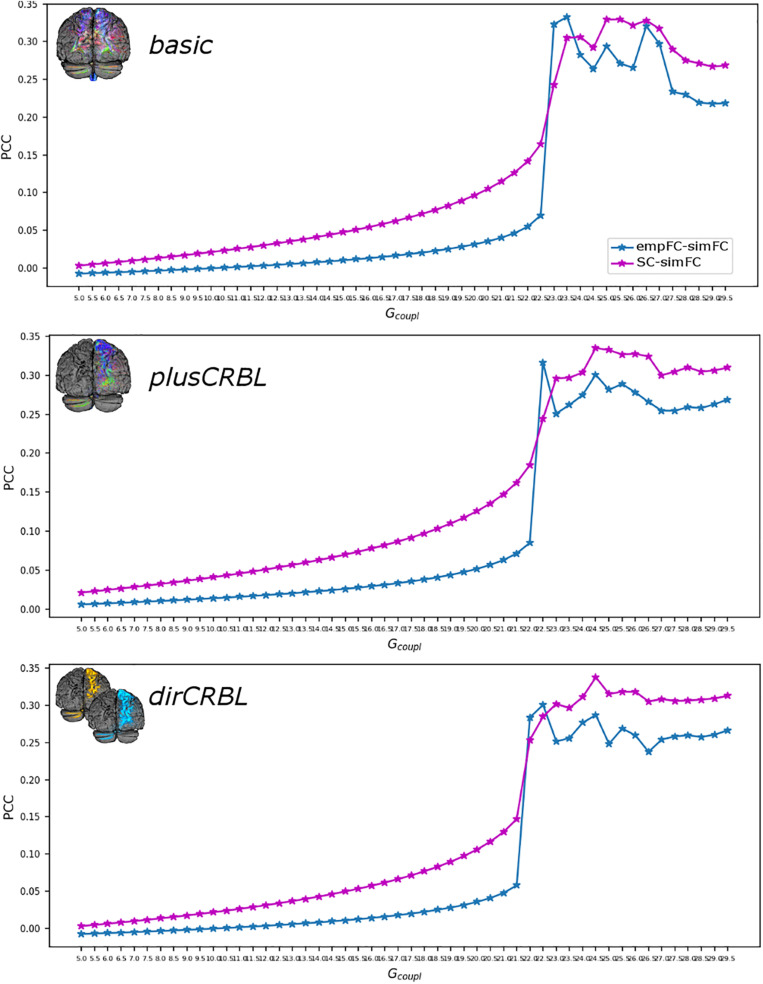
Correlation diagram between empirical and simulated data as a function of the global coupling parameter (G_*coupl*_). The data are taken from a randomly chosen subject for each SC construct (top to bottom: *basic, plusCRBL, dirCRBL*). The correlation between structural and simulated FC is shown with magenta line, while correlation between empirical FC and simulated FC is shown with blue line.

The *G*_*coupl*_ parameter search space showed an increment of the correlation between empFC and simFC toward a single optimal point, which was the absolute maximum of the PCC plot as a function of *G*_*coupl*_ (for greater *G*_*coupl*_ values PCC values were slightly lower but stable). The shape of this curve representing PCC did not differ across SC constructs (see Figure 4 for an example). Furthermore, to assess whether SC *accuracy* could affect model optimization, *G*_*coupl*_, *H*_*low*_, and *H*_*high*_ were compared between SC constructs. Mean across-subject parameters and p-values are reported for each SC construct in Table 2. No differences were found between firing rates, or *G*_*coupl*_ between the SC constructs.

**TABLE 2:** Network and model parameters.

	Basic	PlusCRBL	dirCRBL	*p*-Value
		
	Mean (SD)	Mean (SD)	Mean (SD)	
G_*coupl*_	23.3 (0.95)	22.9 (0.97)	22.6 (1.21)	0.142
H_*low*_ (spikes/s)	100.6 (3.8)	101.3 (5.4)	100.8 (4.5)	0.853
H_*high*_ (spikes/s)	101.3 (3.9)	101.5 (5.2)	101.0 (4.6)	0.918

*Model parameters for the three SC constructs: basic, plusCRBL and dirCRBL. Values are expressed as mean (SD). G_*coupl*_, global coupling; H_*low*_, low firing rate; H_*high*_, high firing rate. *p*-values do not show statistically significant differences between the three SC constructs.*

### Prediction Power of TVB

**FIGURE 5:**
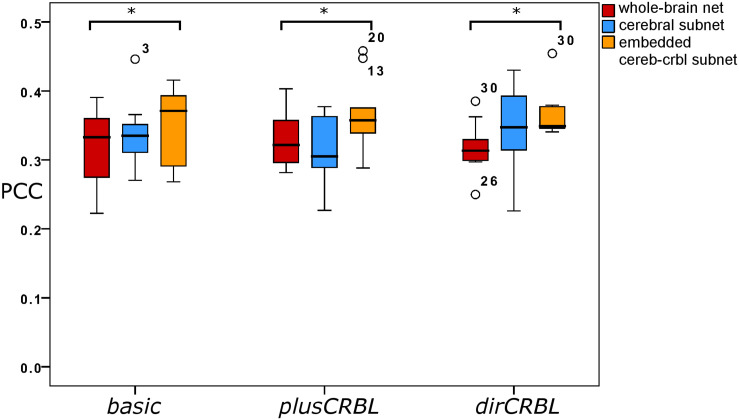
Boxplots of Pearson correlation coefficients for *whole-brain network*, *cerebral subnetwork*, and *embedded cerebro-cerebellar subnetwork* models within each SC construct. Brackets and asterisks indicate significant differences with *p* < 0.002.

In order to examine whether SC *accuracy* or different networks (the SC construct or the embedding of cerebellar SC, respectively), could affect the TVB predictive power of brain dynamics, *similarity* was compared across SC constructs (*basic, plusCRBL, dirCRBL*) and networks (whole-brain network, cerebral subnetwork, embedded cerebro-cerebellar subnetwork). Mean across-subject *similarity* measures between empFC and simFC are reported in Table 3. All networks generated acceptable PCC values (range 0.2224–0.4581) per subject and per SC construct. No differences were observed between SC constructs, while PCC was significantly higher for the embedded cerebro-cerebellar subnetwork than for the whole-brain network within each SC construct (*p* < 0.002). Boxplots of *similarity* measures (Figure 5) show that whole-brain network has the lowest PCC, cerebral subnetwork has an intermediate one and embedded cerebro-cerebellar subnetwork has the highest PCC within *basic* and *dirCRBL*, while the lowest PCC is for the cerebral subnetwork within *plusCRBL* construct.

**TABLE 3:** Pearson correlation coefficients between empirical and simulated FC.

	Basic	plusCRBL	dirCRBL	*p*-Value (between SC constructs)
		
	Mean (SD)	Mean (SD)	Mean (SD)	
Whole-brain network	0.3212 (0.0519)	0.3319 (0.0431)	0.3180 (0.0367)	0.535
Cerebral subnetwork	0.3387 (0.0468)	0.3113 (0.0553)	0.3458 (0.0596)	0.208
Embedded cerebral subnetwork	0.3540 (0.0521)	0.3667 (0.0520)	0.3637 (0.0345)	0.719
*p*-value (between networks)	0.118*	0.049*	0.036*	

Across-subject mean Pearson correlation coefficients between empirical and simulated FC for the three SC constructs (*basic, plusCRBL, dirCRBL*) in the three networks: *whole-brain network, cerebral subnetwork, and embedded cerebro-cerebellar subnetwork*. Values are expressed as mean (SD). *p*-values do not show significant differences between the three SC constructs, while show differences between networks. *p < 0.002 between whole-brain and embedded network. The bold numbers highlight the significative difference between the three groups, identified as the three networks.

## Discussion

The present work provides the first application of TVB extended to cerebellar nodes and cerebro-cerebellar connectivity. The main observation is that the predictive power of the model on brain dynamics is improved by including the cerebro-cerebellar loops. The *similarity* between empFC and simFC significantly increased when cerebro-cerebellar circuits were considered. However, the SC *accuracy*, modified by *a priori* information on cerebro-cerebellar circuits, did not significantly affect either global (whole-brain network) or local (cerebral subnetwork) brain dynamics. This might reflect the small number of cerebro-cerebellar connections compared to the total amount of brain connections. An extended filtering of spurious tracts, which usually affect tractography, would probably allow to improve the precision of predictions on global brain dynamics.

The demonstration of a sizeable contribution of cerebro-cerebellar connectivity to global brain dynamics suggests that TVB has the potential to reveal the impact of specific subnetworks on global brain dynamics and to understand the interaction of multiple brain areas (Aerts et al., 2018; Zimmermann et al., 2018; An et al., 2019). A further improvement to TVB performance could be achieved by using multimodal datasets merging MRI, EEG, MEG, positron emission tomography (PET) data (Schirner et al., 2018; Stefanovski et al., 2019; Triebkorn et al., 2020).

Beside this positive evidence, our result is just a first step toward a mechanistic interpretation of brain dynamics that could be applied to clinical investigations. TVB is an approximation of the brain currently loosing details on microcircuits organization and function. TVB can indeed simulate brain dynamics with some accuracy but without precise correlation with the underlying physiological processes (Ritter et al., 2013; Sanz Leon et al., 2013). Local neuronal microcircuits are characterized by specific structural and functional features and a single model is most likely not be enough to accurately and insightfully simulate whole-brain dynamics (van Wijk et al., 2018; Friston et al., 2019). Using a conservative approach, we assumed that the Wong-Wang model could provide a reasonable approximation of local cerebellar dynamics as it does for the cerebral cortex (Wong and Wang, 2006). However, the microcircuit organization of subcortical structures, especially of the cerebellum, is quite different (D’Angelo et al., 2016). Therefore, specific models reflecting local microcircuit physiology need to be designed and integrated into TVB. This could be achieved by replacing generic oscillatory nodes with specific neural masses or mean field models accounting for local connection rules and specific features reflecting structural and functional knowledge at the microscale. A cerebellar neural mass should include (i) excitatory input units with center-surround organization (representing granule cell clusters) connected in a recurrent inhibitory loop with inhibitory units (the Golgi cells) generating low-frequency oscillations and (ii) perceptrons (the Purkinje cells) connected to a feed forward inhibitory loop (done by molecular layer interneurons). Furthermore, spike-time dependent plasticity rules and geometry-constrained local connectivity (Sgritta et al., 2017) should be defined. These essential computational features have been identified based on experimental and theoretical works on rodents (Marr, 1969; D’Angelo et al., 2016; D’Angelo, 2018). Once this neural mass will be integrated in TVB, microscale data could be used to tune input signals deriving from the long-range structural connectivity based on macroscale empirical information.

Another issue that was opened by our work is how to define the best long-range brain connectivity to be used in TVB. In the MRI field, a relevant limitation is indeed the difficulty to reconstruct crossing and polysynaptic tracts and to identify the direction of axonal propagation (Hein et al., 2016; Nath et al., 2020). We have shown here that connectome corrections integrating physiological and anatomical constraints provide an effective solution that could be extended to an increasing number of long-range brain connections.

## Conclusion

For the first time, TVB simulations show that cerebro-cerebellar connections play an important role in determining whole-brain dynamics. We speculate that future simulations, using either TVB or other similar approaches like DCM, driven by microscale information on specific local microcircuits will increase the reliability of predictions, setting out associations between global signals (like fMRI, EEG, MEG) and “cellular” activity. This will allow to investigate the temporal dynamics of the cerebro-cerebellar circuits within the whole-brain network and to assess their impact on emerging brain functions with special attention to compensatory plasticity and large-scale circuit rewiring as a consequence of various pathologies, which strongly depend on the cerebellum. Moreover, the pipeline presented here could be consistently applied to single patients to predict the course of cerebellar ataxia or psychiatric disorders (i.e., autism), or the progression of neurodegenerative pathologies, or the surgical outcomes, for example, in epileptic patients. This will represent a fundamental step-ahead in understanding physiological and pathological states involving the cerebro-cerebellar circuits.

## Data Availability Statement

The raw data supporting the conclusions of this article will be made available without undue reservation, by contacting the corresponding author.

## Author Contributions

FP, CC, CG, and ED’A conceptualized the study. FP and RL designed and performed the analyses. CG, ED’A, VJ, and PR provided support and guidance with data analysis and interpretation. FP, CG, and ED’A coordinated the project and wrote the manuscript, with comments from all other authors. All authors contributed to the article and approved the submitted version.

## Conflict of Interest

The authors declare that the research was conducted in the absence of any commercial or financial relationships that could be construed as a potential conflict of interest.

## Footnotes

1 http://db.humanconnectome.org

2 https://fsl.fmrib.ox.ac.uk/fsl/fslwiki

3 http://www.mrtrix.org/

4 http://www.nitrc.org/projects/conn

## Abbreviations

BOLD: blood oxygenation level dependent
DCM: Dynamic Causal Modeling
empFC: empirical FC
FC: functional connectome
SC: structural connectome
simFC: simulated FC
TVB: The Virtual Brain.
